# Letter regarding unravelling the genomic landscape of Canadian Borrelia burgdorferi: a comparison across global strains; Piot et al. Microbial Genomics 2026;12:001606

**DOI:** 10.1099/mgen.0.001660

**Published:** 2026-05-11

**Authors:** Nicholas H. Ogden, Samir Mechai, Gabriele Margos, Edward J. Feil

**Affiliations:** 1 Modelling Hub Division, Public Health Agency of Canada, St-Hyacinthe, Canada; 2 National Reference Centre for Borrelia, Bavarian Health and Food Safety Authority, Oberschleißheim, Germany; 3 The Milner Centre for Evolution, University of Bath, Bath, UK

**Keywords:** *Borrelia burgdorferi sensu stricto*, ecology, evolution, Lyme disease

Dear Drs Heinz and Sheppard,

As scientists who have been studying the ecology and evolution of *Borrelia burgdorferi sensu stricto* in Canada (and *B. burgdorferi sensu lato* globally) for some time, we read the above article with interest. Perhaps the study does, for the first time, produce more complete genomes (chromosomes and constructed plasmids) of some Canadian strains by virtue of nanopore long reads, but this is difficult to substantiate, as the reference isolates that were used in the study do not appear complete. While the authors say that they have ‘successfully reconstructed the genome, comprising chromosomes and plasmids of the six Canadian strains’, we have reservations about this statement. Their reconstruction of the B31 genome results in 12 plasmids compared to the 17 to 21 plasmids identified in previous studies [1,2]. As the authors say, *B. burgdorferi* plasmids may be lost in culture, but that may not be the only reason that there are differences in the plasmid content of the different B31 isolates: Margos *et al*. [3] suggested that this may be due to different handling of isolates (such as repeated passage through mice) rather than the loss of plasmids during culture. It also seems possible that there are plasmids missing in other isolates sequenced and assembled by the authors. For example, for isolates 297 and 25015, previous studies found 19 plasmids (297) and 16 plasmids (25015) [2,4] instead of the 15 or 14 plasmids, respectively, identified by Piot *et al*. Furthermore, some plasmids do not seem to be full-length (e.g. cp32, lp56 and lp36). Piot *et al*. use one assembler (Flye v2.9.2) for Oxford Nanopore Technologies reads, when in our experience it is better to use two or three assemblers to ensure assembly of all full-length plasmids [5]. In addition, Unicycler v0.4.8 was used for a hybrid assembly, but this may not properly assemble all plasmids and can introduce errors (in our experience and see [6]). Therefore, while the authors did indeed assemble the chromosome and multiple plasmids for each strain, there is uncertainty as to whether or not these are ‘complete’.

In addition, in our view, the article does not adequately consider the work conducted previously on the phylogeography, ecology and evolution *of B. burgdorferi sensu stricto* in Canada and more widely in North America. The article begins, in the impact statement, with the assertion that ‘little is known about the genetic makeup and evolutionary history of Canadian *B. burgdorferi* strains, limiting our understanding of the pathogen’s origin and adaptation in its new environment’. In fact, we and other colleagues have been studying the genetic makeup and evolutionary ecology of this bacterium in Canada, in particular, and North America, in general, for two decades. The ability to construct plasmids from long reads does increase our toolbox for the study of *B. burgdorferi*, but genotyping methods, such as multilocus sequence typing (MLST) and ospC analysis, used extensively over the past decades, have proven robust and congruent with phylogenies obtained using whole-genome sequencing data [7,8]. Congruence of phylogenies obtained by these methods has been re-emphasized in a more recent study [9]. While the whole-genome sequencing of 64 Canadian strains by Tyler *et al*. [7] did not result in full construction of plasmids (because short-read Illumina sequencing was used), plasmid sequences were sufficient to compare between (and identify congruence of) phylogenies based on chromosomal and global plasmid sequences, as well as plasmid-encoded genes that are frequently used in phylogenetic analyses and strain typing.

Our understanding of the phylogeography and evolutionary ecology of *B. burgdorferi sensu stricto* in North America and specifically Canada is based on much study, in the field, laboratory and *in silico*, and remains unchanged by the study of Piot *et al*. In short, the broad contemporary structure of *B. burgdorferi sensu stricto* populations in North America is constrained by broad longitudinal geographic barriers to gene flow across North America [10]. Phylogenetic patterns are likely shaped by past expansions/contractions of *B. burgdorferi sensu stricto* populations in North America, as well as more recent and current expansions tied to expansions of populations of the tick vector *Ixodes scapularis* and reflecting multiple niche polymorphism, particularly in terms of host specialization [11,15]. Overall, the strains occurring in Canada are likely the same as, or have now evolved from, those in source populations in northern USA, having been carried into Canada in infected ticks carried by northward migrating birds [16,18]. The lack of precise matches of Canadian and US strains is likely due to limitations of the geographic scope of on-the-ground sampling in the less populated areas of the US states bordering Canada, as well as in Canada [18]. Overall, the strain structure in Canada is consistent with south-to-north importation of infected ticks from the USA, with likely impacts of local founder effects [19,20] and geographic patterns of dispersion within Canada [21], with a constant background pattern of geographic range expansion of *I. scapularis* and *B. burgdorferi sensu stricto* driven by climate change [22].

By not situating their results within the context of this established literature, the authors’ interpretations are at times erroneous or overstate the novelty of their findings. For example, the authors say that, based on two strains from the same location not being monophyletic, ‘*B. burgdorferi* strains found in the same Canadian provinces do not have a common geographical origin and appear to have a more complex migration pattern than a simple northward expansion from the northern USA’. Previous studies exploring the population structure in the USA show that multiple clades co-exist in the same broad geographic areas, and the finding of strains that are not monophyletic in a Canadian location to the north of source populations in the USA would not be surprising [18]. Piot *et al*. cite Tyler *et al*. [7] as showing that Canadian strains do not form a monophyletic group and incorrectly conclude that this study shows that ‘the geographical origin of these invading Canadian strains appears to be diverse and mostly unresolved’. In the discussion section of Tyler *et al*., the consistency of the chromosomal tree with the previous MLST-based studies on the evolutionary ecology of *B. burgdorferi* and conclusions on the geographical origin of Canadian strains are described. In the ‘Phylogenetic inference results’ section, there is identification of clustering, or not, of Canadian strains and identification of their MLST and ospC types. These are findings that are not novel to Piot *et al*., having been identified in Tyler *et al*. [7] (see Fig. 2 in that article). The authors state that ‘the topology between phylogenies inferred from eight chromosomal MLST markers and whole chromosome sequences differed significantly and indicated that limited gene marker sets are not representative of the full evolutionary history of the bacteria’. Inspection of the trees in Fig. 3 in Piot *et al*. shows that for the most part, clades are stable across trees (i.e. strains that cluster together remain grouped) and what changes is mainly the relative placement of those clades and a few deeper/internal nodes. That kind of topological discordance at the level of clade ordering is not unexpected, given that the trees are created using different sequences and that the number of sequences used is very small. When using larger data sets, phylogenies using whole genome sequences are concordant with MLST [7,9].

We point out that the Canadian strains used by Piot *et al*., except the single one obtained from the G. Magnotta Lyme Disease Research Lab, University of Guelph, were provided to one of the authors by the National Microbiology Laboratory, Public Health Agency of Canada, and are strains cultured by us from samples we collected in the field for the study published in [7].

While the title of the article suggests that the study comprises ‘a comparison across global strains’, only 13 strains from 5 *B. burgdorferi sensu lato* genospecies are explored when there are 28 known genospecies of this group. In the introduction, it is stated that Lyme disease is emerging in Canada due to the range expansion of the tick vectors *I. scapularis* and *Ixodes pacificus*, but to our knowledge, there is no evidence of the range expansion of *I. pacificus* in Canada.

In summary, we congratulate the authors on developing the methodology presented, but point out limitations to the methods and results. Furthermore, we emphasize the long history of study, in field, laboratory and *in silico*, of the ecology, evolution and phylogeography of *B. burgdorferi sensu stricto* in Canada. Readers of the article should be aware of this literature.

## Abbreviations

MLST: Multilocus sequence typing
